# Supplementary material for: Research of Fluridone’s Effects on Growth and Pigment Accumulation of Haematococcus pluvialis Based on Transcriptome Sequencing
Source: Int J Mol Sci. 2022 Mar 14;23(6):3122. doi: 10.3390/ijms23063122 (PMC8954833; doi:10.3390/ijms23063122)
Supplement: Supplementary file 1 [file ijms-23-03122-s001.zip › ijms-1641893-supplementary.pdf]

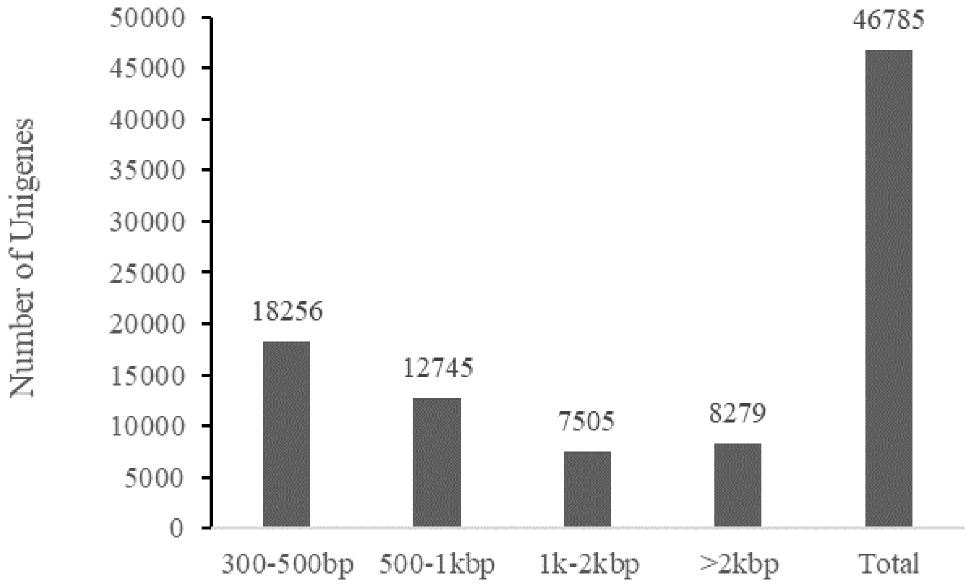

Figure S1. Length interval of unigenes, the abscissa in the figure is the gene length, and the ordinate is the number of transcripts/genes.

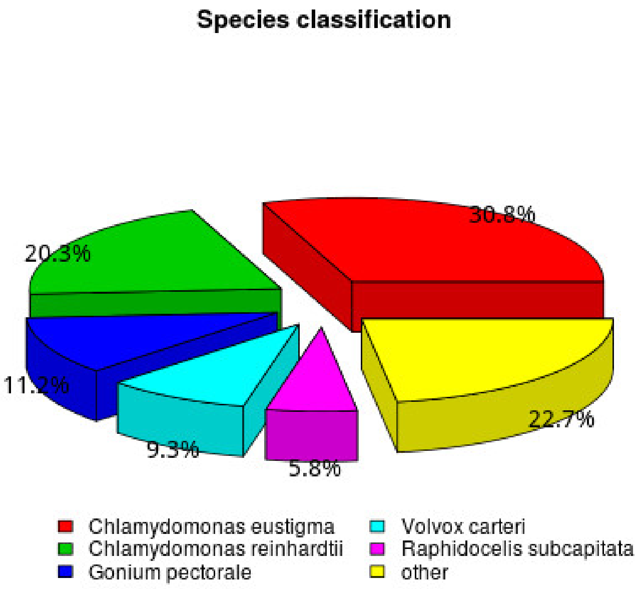

Figure S2. NR library comparison chart.

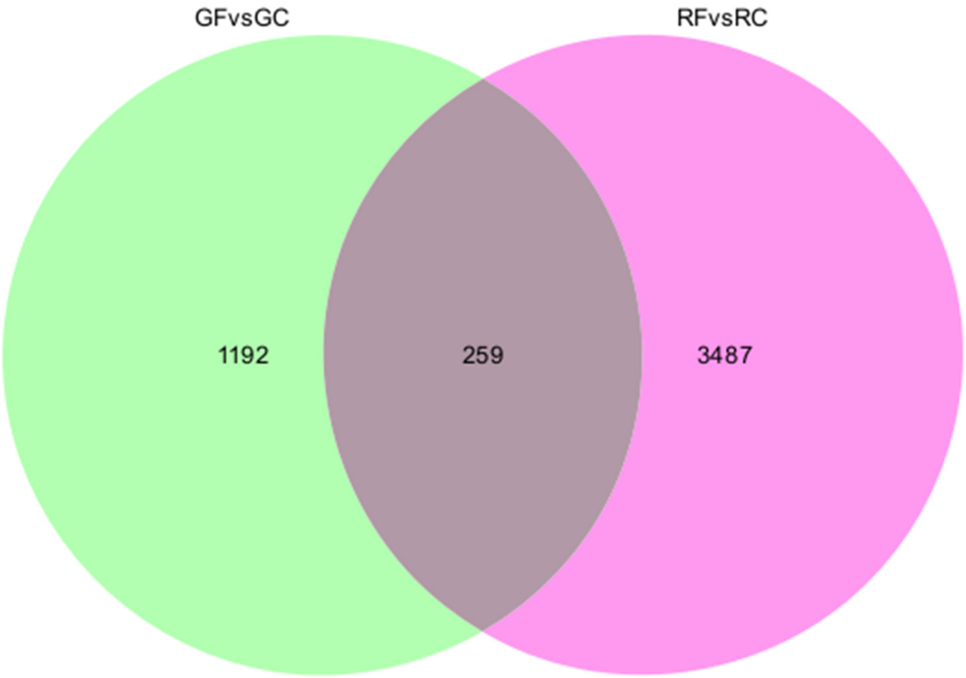

Figure S3. Venn diagram of differentially expressed genes co-expressed in green stage and red stage after fluridone treatment.

Table S1. Statistical display of differentially expressed genes.

Compare	All	Up	Down
GFvsGC	1451	435	1016
RFvsRC	3746	1446	2300
